# Valued life abilities among veteran cancer survivors

**DOI:** 10.1111/hex.12343

**Published:** 2015-01-29

**Authors:** Michele J. Karel, Elizabeth A. Mulligan, Annette Walder, Lindsey A. Martin, Jennifer Moye, Aanand D. Naik

**Affiliations:** ^1^ Mental Health ServiceVA Boston Healthcare SystemBostonMAUSA; ^2^ Department of PsychiatryHarvard Medical SchoolBostonMAUSA; ^3^ Houston VA Health Services Research and Development Center of ExcellenceMichael E. DeBakey VA Medical CenterHoustonTXUSA; ^4^ Section of Health Services ResearchDepartment of MedicineBaylor College of MedicineHoustonTXUSA

**Keywords:** cancer survivors, goal oriented care, older adults, patient centered care, quality of life, values clarification

## Abstract

**Background:**

When patients have multiple chronic illnesses, it is not feasible to provide disease‐based care when treatments for one condition adversely affect another. Instead, health‐care delivery requires a broader person‐centred treatment plan based on collaborative, patient‐oriented values and goals.

**Objective:**

We examined the individual variability, thematic content, and sociodemographic correlates of valued life abilities and activities among multimorbid veterans diagnosed with life‐altering cancer.

**Setting and participants:**

Participants were 144 veterans in the ‘Vet‐Cares’ study who completed a health‐care values and goals scale 12 months after diagnosis of head and neck, gastro‐oesophageal, or colorectal cancer. They had mean age of 65 years and one quarter identified as Hispanic and/or African American.

**Design:**

At twelve months post‐diagnosis, participants rated 16 life abilities/activities in their importance to quality of life on a 10‐point Likert scale, during an in‐person interview. Scale themes were validated via exploratory factor analysis and examining associations with sociodemographic variables.

**Results:**

Participants rated most life abilities/activities as extremely important. Variability in responses was sufficient to identify three underlying values themes in exploratory factor analysis: self‐sufficiency, enjoyment/comfort, and connection to family, friends and spirituality. Veterans with a spouse/partner rated self‐sufficiency as less important. African American veterans rated connection as more important than did White veterans.

**Conclusions:**

It is feasible yet challenging to ask older, multimorbid patients to rate relative importance of values associated with life abilities/activities. Themes related to self‐sufficiency, enjoyment/comfort in daily life and connection are salient and logically consistent with sociodemographic traits. Future studies should explore their role in goal‐directed health care.

Providing the best possible health care for older adults is challenging. Older adults often have multiple chronic conditions, and many face acute episodes of life‐threatening illnesses such as cancer.^1^ Providing the full array of evidence‐based care as defined by clinical practice guidelines for each of these multiple conditions is simply not feasible and may be deleterious when treatments for one condition adversely affect another.^2^ This dilemma is particularly salient for older, multimorbid cancer survivors. The narrow focus on disease categories and the stringent application of targeted therapies with even modest marginal benefit (e.g. initiating a third‐line medication for heart disease when one's pain remains uncontrolled) exacerbates the challenge of addressing functional, social and psychological impairments that cut across multiple conditions and persist for many cancer survivors.^3^ Simply put, using evidence‐based guidelines for individual diseases is an ineffective and potentially harmful strategy when caring for older, multimorbid adults.^1^, ^2^, ^3^

Given this dilemma, clinicians must balance evidence‐based medicine, their understanding of individual health needs, sociocultural context and family dynamics to identify the ‘best available treatment plan’ for a particular patient. In reality, this task is virtually impossible given the time and resource constraints facing most clinicians. Furthermore, many clinicians are not comfortable making the quality‐of‐life trade‐offs required of multimorbidity care without involving patients and caregivers (e.g. what is more important, reducing the discomfort associated with frequent urination at night or the dizziness and risk of falling from the medication used to treat the nocturia?).^4^ The most appropriate strategy for managing older adults with complex impairments is to set collaborative, patient‐oriented goals and identify available treatment strategies for the biopsychosocial challenges that limit goal attainment.^5^

The transition to more patient‐centred health care has inspired many efforts to understand and engage patients in the process of clarifying values and setting goals. The success of goal‐oriented care is predicated on the ability of clinicians, patients and caregivers to articulate which health outcomes are most important and what priority they receive.^6^ Once clear goals are defined and prioritized, clinicians can more effectively work with patients and caregivers on selecting appropriate treatments and refining treatments for goal attainment.^5^, ^6^

Clarifying values, which inform goals of care, is difficult in clinical practice. Individuals vary in their personal values for daily‐life activity, health, longevity and preferences for health care. These differences are influenced by cultural and religious beliefs and traditions, family context, personality and life‐experience.^7^, ^8^, ^9^, ^10^ Scholarship regarding ‘values clarification’ is found in the medical decision‐making literature – concerning a current treatment choice and in advance care planning literature – concerning a range of potential future treatment decisions. In the medical decision‐making literature, values clarification methods help patients clarify and communicate their personal preferences regarding treatment options consistent with their values. Within the context of a specific and current decision, values clarification can improve the match between what is personally most desirable and which option is actually selected, often using decision aides.^11^, ^12^ In the advance care planning literature, values histories and related methods exist to aid patients, caregivers and clinicians to clarify and communicate values that may inform a range of potential, future health‐care decisions.^13^, ^14^, ^15^, ^16^, ^17^, ^18^, ^19^ Methods for conducting values histories include (i) open‐ended interviews, (ii) check lists or rating scales, (iii) evaluation of states worse than death and (iv) narratives (stories and scenarios).^10^ Patients, family members and their clinicians often have difficulty relating personal values to the specific attributes of particular treatment options,^4^ making values clarification challenging to integrate into the workflow of routine clinical care.^11^, ^12^

Values histories are used commonly in advanced directives for end‐of‐life and dementia care, but little is known about their relevance or clinical salience for older, multimorbid adults and especially those planning for survivorship care following cancer treatment. Karel and colleagues^10^, ^15^, ^20^, ^21^ have studied the utility of ‘health‐care values’ tools to help older adults express the aspects of life and functioning that are most important to one's quality of life or that might be most relevant in influencing a potential medical treatment decision. Our team was interested to advance this work by validating the taxonomy of valued life abilities/activities developed in our prior values surveys among a sample of older, multimorbid veterans who are completing treatment for potentially life‐threatening and/or life‐altering cancer.

This study of valued life abilities/activities was nested within the longitudinal Veterans Cancer Rehabilitation Study (Vet‐Cares), which followed a cohort of veterans diagnosed and treated for oral digestive (head and neck, gastro‐oesophageal and colorectal) cancers at 6 (*n* = 170), 12 (*n* = 151) and 18 (*n* = 123) months after cancer diagnosis. At each assessment, veterans engaged in approximately two hours of in‐person interviews that included both structured scales and open‐ended questions regarding their physical, social and psychological experiences of cancer survivorship. On rare occasion, the interview was conducted by phone if necessary and preferred by the veteran. The current nested study describes the valued life ability and activity scale data collected only during the 12‐month interview.

The aims of this analysis were to (i) examine variability and relative ratings of importance of valued life abilities/activities; (ii) explore whether several broad values domains underlie participant responses to valued life ability questions; and (iii) examine the effect of sociodemographic variables on ratings of values domains. A thorough understanding of these aims would facilitate the validation of a values clarification tool for older, multimorbid adults facing the complexities of shared decision making for cancer survivorship.

## Methods

### Recruitment and informed consent

Sample screening and recruitment methodology of the Vet‐Cares study is detailed by Naik *et al*.^22^ In short, eligible participants were identified via regional Veterans Administration tumour registries using diagnosis (ICD‐9 codes for head and neck, oesophageal, gastric, or colorectal cancer) and time of diagnosis (1 month prior to study's opening eligibility window, 6 months) criteria. Veterans with diagnoses of dementia or psychotic disorder were excluded given challenges they might have in completing the study interviews; patients receiving hospice care or considered ‘actively dying’ at baseline assessment were also excluded, as were those with diagnosis of a pre‐cancerous (*in situ*) lesion. Eligible patients were contacted by letter, and then by phone call, to inquire about interest to participate.

Of 639 patients eligible in regard to cancer diagnosis, 223 patients were excluded based on exclusion criteria defined above. Of 416 recruitment letters generated and mailed, 246 patients declined participation. 170 veterans were enrolled in the Vet‐Cares Study for participation in the Time 1 interview, 6 months after diagnosis. At Time 2 (12 months after diagnosis), veterans were re‐contacted with follow‐up letters and phone calls to schedule the next interview. This nested study questions about valued life abilities, and activities were asked only at this Time 2 interview period. Of the 151 Time 2 participants, complete data on the health values and goals scale were available for 144 (95%) individuals.

This study received approval in August 2009 from VA Boston Health Care System and the Baylor College of Medicine/Michael E. DeBakey VA Institutional Review Boards (IRB) (Boston IRB# 2317; Houston IRB # 25446). A partial waiver of written consent allowed screening of cancer registries at both sites. Potentially eligible patients were identified from this screening process and sent an opt‐out letter informing them of the study and that a research coordinator would call to discuss the study unless they chose to opt‐out. Individuals could opt‐out by contacting a toll‐free voicemail line provided in the initial letter or at any point during the subsequent phone call. Patient health information was protected behind the VA electronic firewall, and de‐identified participant identification numbers were used on all subsequent data collection tools and analysis. Veterans completed a written consent form prior to beginning the study, which was entered into the VA Computerized Patient Records System (CPRS) and became part of the patient's permanent medical records. Veterans received $30 compensation for their time after the completion of each interview.

### Variables and measures

#### Demographics

During the Time 1 interview, participants were asked about their gender (male or female), age, education, and race and ethnicity.

#### Cancer type and stage

Cancer type was determined via list of ICD‐9 codes for head and neck, oesophageal, gastric or colorectal cancers. Cancer stage was determined using TNM classification during tumour board report and/or final diagnostic note by oncologist based on the available clinical, radiological and pathology data and confirmed in patients' medical records.

#### Comorbidity Index

Comorbidity ratings used electronic medical record extraction of diagnoses to create a Deyo adjustment of the Charlson Comorbidity Index,^23^ which employs outpatient ICD‐9 data to create an index that predicts 10‐year mortality for a patient who may have a range of comorbid conditions, such as heart disease, AIDS or cancer (a total of 22 conditions). Each condition is assigned a score of 1, 2, 3 or 6, depending on the risk of dying associated with each one. Scores are summed to provide a total score.

#### Religiosity/spirituality

Participants were asked to rate ‘To what degree do you consider yourself a religious person?’ and ‘To what degree do you consider yourself a spiritual person?’ Each item was rated on a 4‐point scale from 0 = not at all to 4 = extremely. These items were closely adapted from recommended single‐item measures of overall self‐ranking of religiosity and spirituality.^24^

#### Social support

Participants were asked whether they have a spouse or partner and how often they have ‘someone you can count on to listen to you when you need to talk?’ and ‘someone to help you if you were confined to bed?’ The latter two items were rated on a 0–4 scale from 0 = none of the time to 4 = all of the time. These items were selected from the Medical Outcomes Study Social Support Survey instrument, as indicators of emotional/information support and tangible support.^25^

#### Health‐care values and goals

The final section of the Time 2 interview was devoted to the topic called ‘your health values and goals’. This section of the interview began with the interviewer saying: ‘We each have different ideas about what makes life most worth living. Experience with serious illness like cancer can lead people to reflect on what is most important in their lives – what are the things in my life that I value the most or that I most want to achieve. In this section, I'd like to ask you to consider which aspects of life are most important to you and how well you are doing in attaining those life goals’.

The *Health Care Values and Goals Scale* was developed for this study, but adapted from earlier work.^21^ The scale was developed in the tradition of ‘values histories’ for advance care planning^14^, ^17^ rather than values clarification tools to help patients make specific medical decisions.^11^, ^26^ The scale items were developed based on literature review regarding advance care planning values histories,^17^, ^18^, ^19^ our prior research related to health‐care values and goals,^4^, ^5^, ^10^, ^20^, ^27^ and investigator consensus on item content relevance for individuals with oral digestive cancers.

Participants were asked to rate 16 ‘life values or goals (i.e. your ability to do these things)’ in two ways. First, they were asked to consider how important each is to their overall quality of life and to rate the item on a visual analog scale from 1 to 10, where the range of 1–2 was defined as ‘Not important to my quality of life; I could live without this’, and 9–10 was defined as ‘Of utmost importance to my quality of life, I could not live without this’. Scale anchors in between were as follows: 3–4 = somewhat important to my quality of life, 5–6 = very important to my quality of life and 7–8 = extremely important to my quality of life.

Of note, we made minor revisions to the scale early in the course of the study, given that participants tended to rate the highest end of the scale, #10, ‘of utmost importance to my quality of life, I could not live without this’ quite frequently. Minor changes were made both to the anchor points on 1–10 scale (i.e. filling in anchor descriptions for points 3–4, 5–6 and 7–8 on the scale) and on the instructions the interviewers used when introducing the scale to the participants. See Supplementary Table 1 for details.

### Analysis

#### Values scale re‐coding

Despite efforts to encourage participants to use the range of the 10‐point scale to rate the relative importance of life abilities/activities, distributions on this scale remained highly skewed to the high end of the scale, with item skewness ranging from −1.84 to −0.56. Approximately one‐third to one half of participants scored ‘10’ for each of the 16 items rated. To create a somewhat more even distribution, we rescored responses on the 10‐point scale to a 4‐point distribution, where scores of 1–5 were rescored as 1; scores of 6 or 7 were rescored as 2; scores of 8 or 9 were rescored as 3 and a score of 10 was rescored as 4 (skew ranged from −1.1 to −0.01 for the transformed variables).

#### Descriptive statistics

Frequency distributions and mean/standard deviations were computed for each item, in terms of importance and achievement.

#### Factor analysis

Exploratory factor analyses guided the development of a taxonomy of broad values domains that underlie participant ratings of the importance of various valued life abilities/activities. The exploratory factor analyses using maximum likelihood estimation with oblique (promax) rotation were conducted in Mplus version 7.^28^ We systematically conducted four‐factor analyses, extracting one, two, three and four factors. Model fit was evaluated using recommendations proposed by Hu and Bentler^29^ for the root mean square error of approximation (RMSEA)^28^ and the standardized root mean square residual (SRMR)^30^ as well as chi‐square difference tests between models.^28^ With the goal of balancing adequate fit indices with the most parsimonious solution, two members of the research team (MK and EM) examined the factor analysis output. Across all of the models, one item did not load clearly on any of the factors (‘To consider the needs and interests of my family’). We removed this item and completed the same analyses with the remaining items. Subscale scores were computed by calculating the mean score of the items loading on that factor.

#### Group comparisons and correlational analyses

To explore the relationship between demographic, social support and religiosity variables and the values subscales, we conducted a series of group mean score comparisons and correlations. We compared the mean score on each subscale for African American and White veterans, and for veterans with and without a reported spouse/partner, using independent sample *t*‐tests. We computed Pearson's product–moment correlations between each subscale score and age, education, extent of social support and religiosity/spirituality.

## Results

### Participants

Table 1 provides descriptive characteristics of the study population. Veteran participants were overwhelming male with a mean age of 65 years. One quarter of the sample was Hispanic or African American, and over 60% were married or had a significant other. Half of the sample had colorectal cancer, and over one‐third had head and neck cancer with an even distribution by cancer stage. The mean comorbidity index score of 6.85 (standard deviation = 4.41) suggests a very high burden of chronic morbidities in this sample. Most prevalent diagnoses after cancer included diabetes (59%), chronic pulmonary disease (36%), peripheral vascular disease (24%) and cerebrovascular disease (15%).

**Table 1:** Characteristics of the study participants

Characteristic	*n* = 144 (%)
Sex	
Male	141 (97.9)
Female	3 (2.1)
Age	
<60	38 (26.4)
60–70	73 (50.7)
>70	33 (22.9)
Ethnicity	
Hispanic, Latino or Hispanic origin?	12 (8.3)
Not	132 (91.7)
Racial identity	
White	115 (79.9)
Black or African American	24 (16.7)
Other or more than one race	5 (3.5)
Spouse or partner	
Yes	89 (62)
No	55 (38)
Education	
Less than high school	21 (14.6)
High school graduate	48 (33.3)
Some college	75 (52.1)
Cancer type	
Colorectal	75 (52.1)
Head and neck	55 (38.2)
Oesophageal/gastric	14 (9.7)
Tumour stage on diagnosis	
1	35 (24.3)
2	42 (29.2)
3	32 (22.2)
4	34 (23.6)
Deyo Comorbidity Index	Mean = 6.85; SD = 4.41

### Health‐care values and goals item endorsement

Table 2 provides the frequency distribution, mean score and standard deviation for the importance attributed to and achievement of 16 life abilities/activities. Items rated on average as most important included the following: to make my own life decisions, to avoid being a burden on others, to have relationships with family and friends and to control my bodily functions. Items rated on average as relatively less important included the following: to practice my religion or spiritual life, to have emotional or sexual intimacy in my life, to engage in productive work and to do specific activities or hobbies that I enjoy.

**Table 2:** Importance of valued life abilities/activities

Life value or goal, that is your ability to do these things:	1 (%)	2 (%)	3 (%)	4 (%)	How important is this to your QOL? *M* (SD)
1. To take care of myself (e.g. bathing, dressing), rather than rely on others for help with daily life	9.0	17.4	34.0	39.6	3.0 (1.0)
2. To walk or move around by myself	8.3	17.4	38.2	36.1	3.0 (0.9)
3. To live at home	8.3	11.8	37.5	42.4	3.1 (0.9)
4. To think clearly about things	10.4	21.5	27.8	40.3	3.0 (1.0)
5. To avoid being a burden to others	6.3	15.3	30.6	47.9	3.2 (0.9)
6. To practice my religion or spiritual life (faith, prayer)	36.8	11.1	19.4	32.6	2.5 (1.3)
7. To have relationships with family and friends	7.6	14.6	29.2	48.6	3.2 (1.0)
8. To make my own life decisions (e.g. about health, finances, housing)	3.5	9.7	34.7	52.1	3.4 (0.8)
9. To have my privacy	11.8	17.4	29.2	41.7	3.0 (1.0)
10. To have emotional or sexual intimacy in my life	22.4	16.8	31.5	29.4	2.7 (1.1)
11. To consider the needs and interests of my family	6.3	17.4	34.0	42.4	3.1 (0.9)
12. To live without significant pain or discomfort	12.5	17.4	27.8	42.4	3.0 (1.1)
13. To be able to eat ‘normally’, to enjoy meals	9.7	16.7	31.9	41.7	3.1 (1.0)
14. To control my bodily functions (e.g. urination)	7.6	12.5	30.6	49.3	3.2 (0.9)
15. To engage in productive work – in a job, at home or in the community	18.2	15.4	35.7	30.8	2.8 (1.1)
16. To do specific activities or hobbies that I enjoy (e.g. reading, TV, gardening)	18.8	12.5	36.8	31.9	2.8 (1.1)

1 = Score of 1–5 on original 10‐point rating scale; 2 = Score of 6 or 7 on original 10‐point rating scale; 3 = Score of 8 or 9 on original 10‐point rating scale; 4 = Score of 10 on original 10‐point rating scale.

Columns may not add to 100% due to rounding.

### Factor analysis: quality‐of‐life values subscales

The three‐factor model was both clearly interpretable and the best‐fitting solution (RMSEA = 0.05; SRMR = 0.04). In addition to having superior fit indices in comparison with the one (RMSEA = 0.09; SRMR = 0.08)‐ and two‐factor solutions (RMSEA = 0.06; SRMR = 0.05), the three‐factor model was a significantly better fit than the 2‐factor model based on the chi‐square difference test, χ^*2*^(13, *n *= 144) = 32.927, *P *<* *0.01. The four‐factor model was inadmissible due to a negative residual variance. Promax‐rotated factor loadings for the three‐factor solution are presented in Table 3. These factors were given the following names using a consensus‐based approach among the research team members: self‐sufficiency (seven items), enjoyment/comfort (six items), and connection (two items).

**Table 3:** Exploratory factor analysis: promax‐rotated loadings

Life value or goal, that is your ability to do these things	1 Self‐sufficiency	2 Enjoyment/comfort	3 Connection
1. To take care of myself (e.g. bathing, dressing), rather than rely on others for help with daily life	**0.741**	−0.166	0.047
2. To walk or move around by myself	**0.630**	0.059	0.139
3. To live at home	**0.658**	0.150	−0.113
4. To think clearly about things	**0.734**	0.043	0.061
5. To avoid being a burden to others	**0.535**	0.152	0.145
6. To practice my religion or spiritual life (faith, prayer)	−0.041	−0.043	**0.614**
7. To have relationships with family and friends	0.174	−0.054	**0.661**
8. To make my own life decisions (e.g. about health, finances, housing)	**0.457**	0.359	−0.021
9. To have my privacy	0.100	**0.817**	−0.282
10. To have emotional or sexual intimacy in my life	−0.093	**0.553**	0.153
11. To consider the needs and interests of my family (Note: Not included in factor analysis)			
12. To live without significant pain or discomfort	0.034	**0.516**	0.241
13. To be able to eat ‘normally’, to enjoy meals	0.106	**0.503**	0.250
14. To control my bodily functions (e.g. urination)	**0.387**	0.301	0.073
15. To engage in productive work – in a job, at home or in the community	−0.083	**0.429**	0.324
16. To do specific activities or hobbies that I enjoy (e.g. reading, TV, gardening)	0.054	**0.591**	0.073

Numbers in bold indicate the relatively highest factor loading for each scale item. Subscales were determined accordingly.

Subscale scores were computed by calculating the mean score of the items loading on each factor. Internal consistency of the subscales was good for self‐sufficiency (Cronbach's α = 0.87) and enjoyment/comfort (Cronbach's α = 0.81), but lower for the 2‐item connection subscale (Cronbach's α = 0.57). The subscales were significantly intercorrelated (*r =* 0.63 for self‐sufficiency and enjoyment/comfort; *r = *0.39 for self‐sufficiency and connection; *r = *0.45 for enjoyment/comfort and connection, all *P *<* *0.001).

### Sociodemographic and clinical correlates of valued life abilities/activities

Veterans who reported having a spouse or partner had lower mean importance ratings on the self‐sufficiency subscale than did veterans who reported having no spouse or partner [*M* = 3.00, SD = 0.65 vs. *M* = 3.36, SD = 0.71, respectively; *t* (d.f. = 142) = 3.11, *P *=* *0.002].

African American veterans (*n* = 24) had higher mean importance ratings on the connection subscale compared to White veterans [*M* = 3.21, SD = 0.95 vs. *M* = 2.77, SD = 0.93, respectively; *t* (d.f. = 137) = 2.08*, P *<* *0.05]. On an item level, this difference was attributable to Black veterans rating being able to practice religion or spiritual life as more important, on average, than did White veterans.

Veterans with higher self‐reported religiosity or spirituality tended to have higher importance ratings on the connection subscale (*r *=* *0.37 and *r *=* *0.42, respectively, *P *<* *0.001 in both cases). Veterans who rated themselves as more spiritual also had higher importance ratings on the enjoyment/comfort subscale (*r *=* *0.20, *P *<* *0.05).

Veteran age, education and extent of reported social support did not relate to importance ratings of valued life abilities/activities.

## Discussion

In contrast with disease‐oriented care which aligns individual treatments to single conditions,^6^ goal‐oriented care is the alignment of health care (therapeutic options) to meet collaboratively set goals that are relevant regardless of specific chronic and acute conditions. Older veterans, who are typically multimorbid, require a patient‐centred approach that includes collaborative goal setting involving their clinicians and families/caregivers. The prioritization of health‐care values and related goals may evolve over time due to the changing physical and mental capacities of older adults and the dynamics of their caregiving relationships.^4^, ^31^ Challenges in goal‐oriented care relate in part to a gap in understanding how values for life activities and abilities shape patients' selection of treatments and prioritization of health goals.

Values and goals are deeply rooted in human biology, psychology and sociocultural systems. At their foundation, individual health‐care goals are cognitively and affectively informed by broad personal values that guide individual decisions towards biological homeostasis^32^ and psychological flourishing^33^ and collectively towards sociocultural cohesion and development.^34^ The values that form the foundation for our health and health‐care goals most likely relate to domains of life functioning, perceived benefits and burdens of treatment options, and how health affects our sense of identity, family, culture and spirituality/religion. This realm of values and goal‐oriented care drifts far from the training and experiences of clinicians, enculturated in biomedicine and a focus on acute illness and guideline‐driven care.

In the context of this clinical milieu, the current study sought to explore life and health values of older, chronically ill veterans who were facing significant threats to mortality and quality of life due to a recent diagnosis and treatment for cancer (head and neck, gastric/oesophageal or colorectal). With increased survivorship, cancer is now often considered not terminal but instead an additional chronic illness whose treatments also may create chronic conditions that add to overall multimorbidity. Study participants completed a scale asking them to rate the level of importance of each of 16 valued life abilities/activities to their quality of life. While participants were able to respond to scale items during the research interviews, they did have difficulty prioritizing among 16 valued life abilities. Consistent with previous work,^10^, ^21^ they rated many items at the highest point of the 10‐point scale, that is, ‘Of utmost importance to my quality of life; I could not live without this’. Most of us cannot imagine living without some degree of independence, bodily functioning and capacity to engage in valued activities and relationships. Therefore, it is not surprising that these valued life abilities were rated as very important by most participants, even with great effort to encourage them to prioritize responses on a 1–10 point rating scale. It is difficult to imagine, even for people going through treatments for cancer, what life might be like without certain basic abilities; people do, however, adapt quite well and shift their sense of what is most important when abilities do change.^31^, ^35^, ^36^

Despite this skewed response to the values items, this study validated three meaningful values domains via a factor analysis: self‐sufficiency, enjoyment/comfort and connection (interpersonal and/or spiritual). These domains were associated with several sociodemographic characteristics in expected ways and are consistent with those identified in our previous and other studies.^10^, ^20^, ^21^, ^37^, ^38^ Depending on the scales and how questions are framed, values related to medical decision making include autonomy/self‐sufficiency, pain/physical comfort, ability to communicate, concerns for being a burden (physical, financial, emotional), concerns for impact on family/family relations, preservation of life, maintaining quality of life and preferred involvement in decision making.

In the current health‐care environment that encourages quality and value over fee‐for‐service reimbursement, patients will increasingly need to make choices about what aspects of functioning are of greater value or importance to them. These choices will help guide clinical decision making towards a more patient‐centred orientation. For example, patients with cancer may choose lower cost supportive medications and services that would increase their comfort and ability to remain at home in place of costly additional chemotherapy that would provide only modest improvements in morbidity and mortality.^39^ The results of this study offer a patient‐centred context (health values and goals) for guiding discussions of treatment planning and prioritization. While patients may have difficulty explicitly prioritizing or ranking values, it may be useful to inquire of, or cue, patie‐nts about values domains that may relate to their weighing of treatment options.^40^ In addition to decision aides designed to help patients consider the risks and benefits associated with a particular treatment decision,^11^, ^41^ it may be helpful to ask patients to share their perspectives on self‐sufficiency, comfort/enjoyment and connection in their lives and to consider how current treatment decisions may influence those life domains.

For example, regarding self‐sufficiency, clinicians may ask patients to reflect on concerns they have about how their illness/treatments may affect independence and functioning in everyday activities. Regarding comfort/enjoyment, clinicians may ask about what makes life meaningful and enjoyable, what might make life feel unbearable and what is most important in terms of everyday comfort and symptom management. Regarding connection, it can help to ask about important relationships and religious/spiritual practices, and concerns about the impact of illness/treatments on those connections; for example, what worries might a patient have about the impact of cancer, for example, on relationships (e.g. difficulty helping loved ones, reduced intimacy, posing an emotional or financial burden)? It is important to note that some patients may prefer to defer complex medical decision making to trusted health‐care providers and/or family members, and that, too, is a value to be respected.

These analyses do have limitations worth noting. The limited number and framing of values domains in this study (self‐sufficiency, comfort/enjoyment and connection) may not reflect all the values domains important to older morbid adults when making health‐care decisions. In this analysis, we focused on abilities influencing quality of life and did not ask participants to rate other values that might influence health‐care decisions, such as relative emphasis on maintaining quality vs. quantity of life or preferences for extent of participation in decision making.^9^, ^16^ We had relatively few questions that tapped the domain of connection, which likely accounts for the lower internal consistency (Cronbach's alpha) for this domain. The connection domain can certainly be elaborated in terms of the range of values people hold about their relationships (to people, higher power, pets, etc.) and the impact of illness on these connections, that is the extent to which values regarding relationships, and impact of health‐care decisions on loved ones, may influence health‐care decision making. In addition, the elaboration of scale response options and changes in interviewer instructions early in the study to address skewed responses may modestly affect results. However, in the end, we did not find significant differences in mean endorsement of values at both the subscale and item level across the three versions of interviewer instructions. The findings of this study may be limited to mostly male US military veterans facing health‐care decisions related to cancer survivorship and may not be generalizable to all multimorbid older adults or all cancer survivors, nor to females in these groups.

This study demonstrated both the feasibility and challenge of asking older patients with complex medical conditions to rate the relative importance of their life values/goals. Further research is needed to confirm the relevance of the values domains identified in this study in other clinical populations. In this sample of mostly older male veterans with cancer and other chronic medical illnesses, responses may reflect specific cohort, gender or illness‐related priorities. Likewise, research is needed to identify additional values domains that may not have been identified in this study. Finally, while this study represents an important step towards understanding how to elicit patients' values, additional research is needed to identify methods for integrating such values clarification processes into shared decision making.^12^ Additional research is needed to elaborate the context and process of patient‐centred discussions of valued life activities/abilities and how these discussions can enhance the process of goal‐oriented care.

## Funding

Funding for this study was provided by the Department of Veterans Affairs Rehabilitation Research and Development Service (5I01RX000104; PI: Moye).

## Conflict of interest

The author(s) declares that they have no competing interests.

## Supporting information


**Table S1**. Adaptations to the health care values and goals scale instructions and anchors.
