# Supplementary material for: Valued life abilities among veteran cancer survivors
Source: Health Expect. 2015 Jan 29;19(3):679–90. doi: 10.1111/hex.12343 (PMC4869069; doi:10.1111/hex.12343)
Supplement: Supplementary file 1 — Table S1. Adaptations to the health care values and goals scale instructions and anchors. [file HEX-19-679-s001.docx]

**Supplemental Table 1. Adaptations to the Health Care Values and Goals Scale Instructions and Anchors**

| Revision date | Introductory narrative | Scale anchors | Scale instructions | # participants who responded to this version |
| --- | --- | --- | --- | --- |
| April 2010, original | We each have different ideas about what makes life most worth living. Experience with serious illness like cancer can lead people to reflect on what is most important in their lives – what defines a good “quality of life.” In this section, I’d like to ask you to consider which aspects of life are most important to you. | 1 = Not important to my quality of life: I could easily live without having this ability  10 = Of utmost importance to my quality of life: life would be unbearable if I no longer had this ability | Many of these things may be important to you. But some will be more important than others.  Please take your time and reflect on their relative importance, using the entire scale from 1-10. | Boston: 5  Houston: 13 |
| October 2010 | We each have different ideas about what makes life most worth living. Experience with serious illness like cancer can lead people to reflect on what is most important in their lives – what are the things in my life that I value the most or that I most want to achieve. In this section, I’d like to ask you to consider which aspects of life are most important to you and how well you are doing in attaining those life goals. | 1-2 = Not important to my quality of life; I could live without this.  3-4 = Somewhat important to my quality of life  5-6 = Very important to my quality of life  7-8 = Extremely important to my quality of life  9-10 = Of utmost importance to my quality of life; I could not live without this. | First, I want you to look over the entire list of values and goals and rate how important each is to your overall life. I want you to select the one that you would rate the highest above all other, think “I could not live without this one.” Please rate that goal a 10.  Next, I want you to select the goal or value that is of least importance to you. You would rate this one lower in importance than all the others. Look at the scale below and give it an appropriate rating.  Then, look at the remaining list of goals and values and rate each in comparison to your highest and lowest rated goals. | Boston: 5  Houston: 10 |
| December, 2010 | Same as above | Same as above | Same as above, with prompt added:  Prompts:  *If veteran rates one item as a 10, then later rates another item a 10, say “*Is this as equally as important as your first 10. If it’s not, then maybe you should think about another number.” | Boston: 40  Houston: 71 |
